# Targeting Angiogenesis and Tumor Microenvironment in Metastatic Colorectal Cancer: Role of Aflibercept

**DOI:** 10.1155/2014/526178

**Published:** 2014-07-21

**Authors:** Guido Giordano, Antonio Febbraro, Michele Venditti, Serena Campidoglio, Nunzio Olivieri, Katia Raieta, Pietro Parcesepe, Giusy Carmen Imbriani, Andrea Remo, Massimo Pancione

**Affiliations:** ^1^Medical Oncology Unit, Ospedale Sacro Cuore di Gesù Fatebenefratelli, 82100 Benevento, Italy; ^2^Department of Biology, Federico II University, 80131 Napoli, Italy; ^3^Department of Sciences and Technologies, University of Sannio, 82100 Benevento, Italy; ^4^Department of Surgical and Diagnostic Pathology, “G.B. Rossi” Hospital, University of Verona, 37134 Verona, Italy; ^5^Fifth Division of General Surgery and Special Surgical Techniques, Second University of Studies of Naples, 80138 Naples, Italy; ^6^Department of Pathology, “Mater Salutis” Hospital, 37045 Legnago, Italy

## Abstract

In the last decades, we have progressively observed an improvement in therapeutic options for metastatic colorectal cancer (mCRC) treatment with a progressive prolongation of survival. mCRC prognosis still remains poor with low percentage of 5-year survival. Targeted agents have improved results obtained with standard chemotherapy. Angiogenesis plays a crucial role in colorectal cancer growth, proliferation, and metastasization and it has been investigated as a potential target for mCRC treatment. Accordingly, novel antiangiogenic targeted agents bevacizumab, regorafenib, and aflibercept have been approved for mCRC treatment as the result of several phase III randomized trials. The development of a tumor permissive microenvironment via the aberrant expression by tumor cells of paracrine factors alters the tumor-stroma interactions inducing an expansion of proangiogenic signals. Recently, the VELOUR study showed that addition of aflibercept to FOLFIRI regimen as a second-line therapy for mCRC improved significantly OS, PFS, and RR. This molecule represents a valid second-line therapeutic option and its peculiar ability to interfere with placental growth factor (PlGF)/vascular endothelial growth factor receptor 1 (VEGFR1) axis makes it effective in targeting angiogenesis, inflammatory cells and in overcoming resistances to anti-angiogenic first-line treatment. Here, we discuss about Aflibercept peculiar ability to interfere with tumor microenvironment and angiogenic pathway.

## 1. Introduction

Colorectal cancer (CRC) represents the third most common cancer worldwide with 1,200,000 newly diagnosed cases each year and the second leading cause of cancer related deaths with 600,000 deaths annually [1, 2]. Nowadays overall survival in metastatic patients has reached approximately 24 months, mostly thanks to the introduction of targeted agents who have improved efficacy of standard chemotherapy. For more than fifty years, 5-fluorouracil has represented the backbone of all chemotherapy schedules, used both alone and combined. The addition of oxaliplatin and irinotecan to fluorouracil based treatment has increased response rate (RR) and overall survival (OS) [3–5]. Nevertheless, chemotherapy alone reached 18–20 months survival plateau obtained administering alternatively all active cytotoxic agents in the course of treatment strategy [6]. Genetic and molecular studies have led to better understanding of tumor growth, proliferation, differentiation, and metastasization pathways, resulting in the development of molecular targeted agents. Two major pathways are involved in mCRC: the epidermal growth factor receptor (EGFR) cascade and the vascular endothelial growth factor (VEGF) signaling [7]. Randomised phase III clinical trials have shown that targeting EGFR with monoclonal antibodies cetuximab and panitumumab has resulted in improved outcomes in mCRC patients with the limitation to Ras wild-type population [8–10]. On the other hand, given the crucial importance of neoangiogenesis in mCRC, anti-VEGF pathway therapies have been intensively investigated [11]. Monoclonal antibody bevacizumab is the first antiangiogenic agent to be approved in mCRC treatment, because of response rate and survival benefit [12]. Recently, new antiangiogenic agents, aflibercept and regorafenib, with mechanisms of action different from bevacizumab, have increased the survival in mCRC patients [13, 14].

Growing evidence suggests that the crosstalk between genomic/epigenomic aberrations of malignant cells and the surrounding microenvironment that is composed of immune and stromal cells contributes to the enhancement of tumor growth culminating in metastatic dissemination [15–17]. Over the past decades, it has become increasingly clear that the mobilization of immune cells, such as myeloid derived suppressor cells (MDSCs) and tumor associated macrophages (TAMs) may contribute either to a lack of response or to acquired drug resistance [18]. Recent studies have shown that vascular endothelial growth factor A (VEGF-A) signaling through vascular endothelial growth factor receptor-2 (VEGFR-2) is involved in MDSCs precursors recruitment to metastases and, once within the tumor, MDSCs can further mature into tumor-promoting macrophages. Additional angiogenic factors such as the placental growth factor (PlGF) directly or indirectly stimulate angiogenesis by affecting a wide range of different cell types or by attracting MDSCs and macrophages within the tumor microenvironment (TME). Notably, PlGF activates inflammation and pathological angiogenesis mainly by interacting with alternative pathways via VEGFR-1 signaling. This is a relevant issue given that myeloid cells primarily express VEGFR-1 and not VEGFR-2 and PlGF exerts complementary effects, independent of VEGF-A [19, 20]. Myeloid cells have been considered the main mechanism of resistance to anti-VEGF targeted therapies by secreting additional proangiogenic factors [21, 22]. These observations suggest that the cells surrounding the tumor microenvironment expand angiogenic signaling pathways through from VEGF-A; this condition seems to be crucial for tumor outgrowth, regrowth, or metastatic dissemination [23, 24].

In this paper, we focus our attention on angiogenesis and its dialogue with TME and antiangiogenic therapies in mCRC, underlining the role of aflibercept and its possible employment in clinical practice.

## 2. Tumor Angiogenesis and Its Mediators

**Figure 1:**
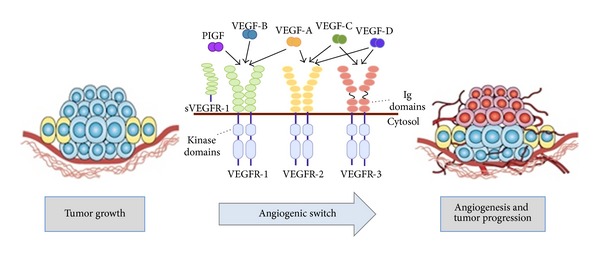
Angiogenesis promotes cancer growth and metastasis. Angiogenesis, the process of developing new blood vessels from preexisting vascular networks, is a well-described mechanism leading to the initiation and maintenance of tumours and the promotion of metastasis at secondary sites. The vascular endothelial growth factor (VEGF) family of ligands and receptors mainly includes VEGF-A, VEGF-B, VEGF-C, VEGF-D, placental derived growth factor (PlGF), VEGFR-1, VEGFR-2, and VEGFR-3. The best characterized of the VEGF family members is VEGF-A, whose binding to VEGFR-2 (FLK1) is the predominant mechanism through which tumour cells promote the so-called angiogenic switch.

**Figure 2:**
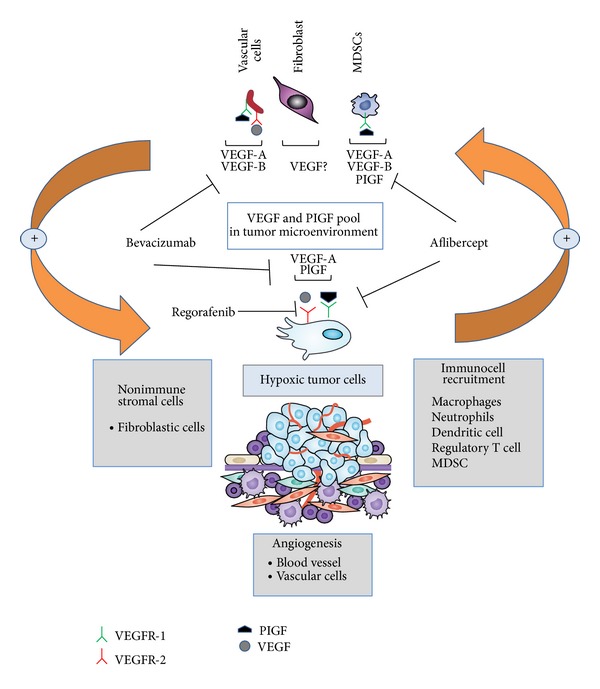
New molecules targeting angiogenesis and crosstalks between angiogenesis and tumor microenvironment. Simplified and schematic view of how multiple cells contribute to the VEGF and PlGF pool in the tumor microenvironment. The tumor microenvironment (TME) consists of soluble molecules, immune, nonimmune fibroblastic, vascular, and malignant cells that interact in a paracrine and autocrine fashion to promote cancer growth and metastasis. Hypoxia is the most potent stimulus for inducing the main angiogenic factors, VEGF and PlGF. Myeloid derived suppressor cells (MDSCs) might confer resistance to therapies that target VEGF by secreting additional proangiogenic factors and specifically by expressing VEGFR-1 (also known as FLT1). PlGF signals directly through VEGFR-1 in various cell types, including endothelial cells, angiogenesis-competent myeloid progenitors, macrophages, and tumour cells and thereby promotes tumour growth and the formation of the premetastatic niche. A substantial fraction of tumours is resistant or escapes antiangiogenic inhibitors that target VEGF-A signalling (bevacizumab) through therapy-induced injury, metabolic changes, inflammation, and possibly expansion of MDSCs. Differently from other antiangiogenic drugs, aflibercept targeting PlGF should reduce the source of the compensatory upregulation of angiogenic factors by inhibiting immune cells recruitment and/or polarization and the release of angiogenic factors by tumour and vascular cells. Regorafenib is a multikinase inhibitor against selected tyrosine kinases and signal transduction VEGFR2-3/RAF/MEK/ERK pathway.

Angiogenesis is defined as the process of blood vessels neoformation through a multistep mechanism that provides nutrients and oxygen to tissues, allowing discharge of waste products [25]. This process works in physiological conditions such as wound healing, embryogenesis, and inflammation, but it is also crucial during pathological conditions like cancer [24]. Angiogenesis is a well-regulated process and, in normal conditions, there is a balanced equilibrium between pro- and antiangiogenic factors as well as between multiple signalling pathways [26, 27]. Under conditions of malignancy, like during neoplastic processes, there is a disruption of the balance between proangiogenic and antiangiogenic factors better known as “angiogenic switch” driven by the increase of nutrient supply necessary for tumor growth (Figure 1) [28, 29]. VEGFs represent the most important and widely studied proangiogenic factors family and it is composed of 5 growth factors named VEGF-A, VEGF-B, VEGF-C, VEGF-D, and PlGF [30–32]. VEGFs play their role in angiogenesis through binding to three different receptors located on cell membrane: VEGFR-1 (Flt-1), VEGFR-2 (Flk/KDR), and VEGFR-3 (Flt-4) (Figure 1). VEGFRs structure consists of an extracellular domain that binds specific ligands, a transmembrane domain, and an intracellular region with tyrosine kinase domain [33]. Activities of VEGFRs can be enhanced by neuropilin receptors (NRP-1 and NRP-2) that may act like coreceptors for VEGFs [34]. VEGF-A represents the first member of VEGF family and the most intensively studied. It is also the most important regulator in human physiologic and pathologic angiogenesis and it is related to a poor prognosis in several cancers [35]. VEGF-A may interact with both VEGFR-1 and VEGFR-2, but the major VEGFR-2 intrinsic kinase activity makes it the most important effector of VEGF-A downstream signalling [36]. The effects of this interaction are represented by endothelial cell proliferation, survival, migration, invasion, vascular permeability, and vasodilatation. Less is known about the other members of VEGF family. VEGF-B has common structural homology with VEGF-A and its activity, mediated by interaction with VEGFR-1 and NRP-1, could have a role in tumorigenesis and blood vessels survival under stress conditions [37]. VEGF-C and VEGF-D that bind VEGFR-3 are involved in lymphangiogenesis. In particular VEGF-D expression has been associated with tumor metastasization to regional lymph nodes [38]. PlGF is another important growth factor which regulates vessel growth and maturation directly by affecting endothelial and mural cells, as well as indirectly by recruiting proangiogenic cell types (Figure 2). PlGF shares structural homology with VEGF-A and stimulates angiogenesis via interaction with VEGFR-1 downstream signaling [39–42] (Figure 1). In fact, PlGF binding to VEGFR-1 modulates recruitment of bone marrow derived macrophages to the tumor site, where they release proangiogenic factors [43]. There are many evidences that PlGF and VEGFs levels are strongly involved in CRC proliferation and metastasization influencing patients outcome [44–47]. Activation of proangiogenesis factors and/or microvessel density has been related to increased vascularization, advanced disease, and poor prognosis in a variety of tumours [48, 49]. It has been demonstrated that there is a strong correlation between vascular density in primary colorectal cancer recurrence, metastasis, and mortality [50]. Unlike VEGFA, which binds to both VEGFR-1 and VEGFR-2, PlGF binds to VEGFR-1 but not VEGFR-2 (Figure 1). Several reports have documented that also PlGF and VEGFR-1 are increased in various tumors, which correlate with disease progression and can predict poor prognosis, metastasis, and recurrent disease [51–53]. The concept of angiogenesis as a marker of tumor aggressiveness is enforced by evidences of higher vascular density at the tumor invasion front if compared to other areas within the tumor [53]. Furthermore, an increased expression of VEGF mRNA has been detected in human CRC liver metastasis, and VEGFR-1 and VEGFR-2 expression were upregulated in liver metastases compared with contiguous normal liver [54–56].

## 3. Relation between Tumor Microenvironment and Angiogenesis

**Figure 3:**
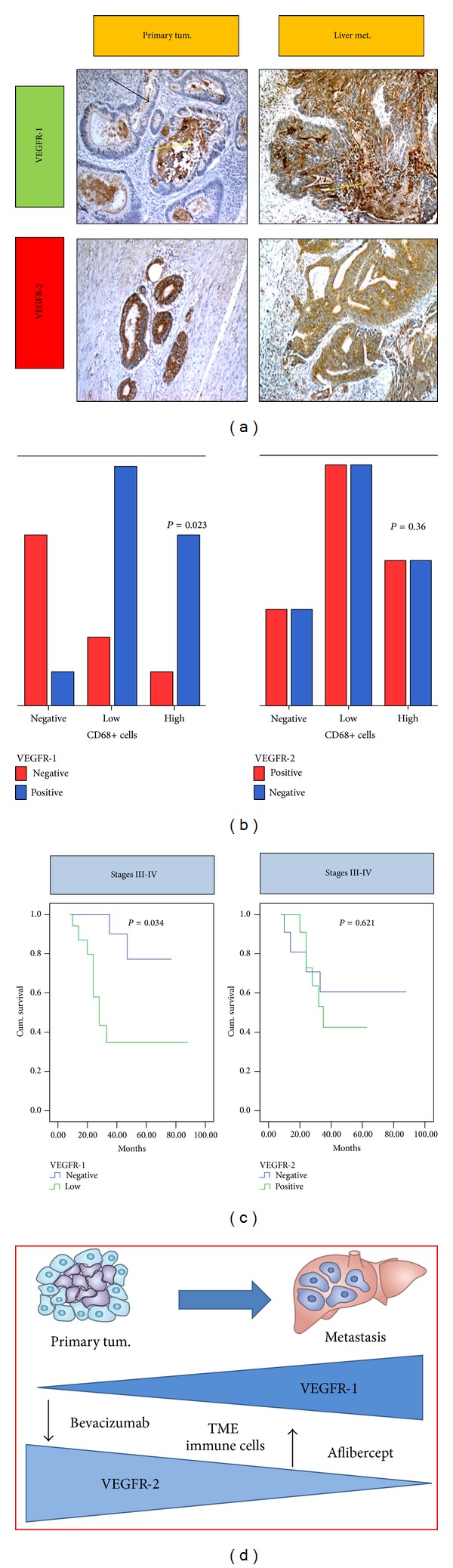
VEGFR-1 and VEGFR-2 differentially orchestrate the tumour-stroma interplay to promote cancer growth and metastasis. (a) Representative images of VEGFR-1 and VEGFR-2 immunostaining in a primary colon carcinoma and corresponding liver metastasis obtained from our still unpublished observations. Yellow and black arrows indicate the immunostaining in the stromal compartment and malignant colonic cells, respectively; with magnification 10x. (b) VEGFR-1 and VEGFR-2 immunostaining differentially correlate with CD68 infiltration, a marker of M2 tumor associate macrophages (TAMs). (c) Patients' disease specific survival in relation to VEGFR-1 and VEGFR-2 expression in our cohort of 86 CRC patients stage III-IV only (Pancione et al. unpublished data). (d) Schematic drawing of the proposed mechanism(s) involved in metastasis-promoting actions of VEGFR-1 and VEGFR-2, respectively. VEGFR-1, but not VEGFR-2, is expressed in a variety of stromal cells and appears synergized with TME in the evolution of premetastatic niche and cancer cell migration. Aflibercept targeting PlGF/VEGFR-1 axis can reduce the induction of an angiogenic rescue program and inhibit immune cells recruitment and metastatic progression. The *P* value is reported in each graph. Cum. (cumulative), Met. (metastases), Neg. (negative), Pos. (positive), and Tum. (tumor).

A general feature of advanced tumors is represented by the altered reciprocal interaction of cancer cells with surrounding nonmalignant cells, soluble inflammatory mediators, and components of the extracellular matrix. Emerging evidence suggests that the interconnected plasticity of tumor cells and stromal cells in response to treatment-induced tumor tissue injury and inflammation represents a common aspect of therapy resistance. One of the mechanisms that tumors utilize to promote metastases and escape antiangiogenic therapies is the activation of inflammatory cells and MDSCs (Figure 2). Consistently, our results (Pancione et al. unpublished data) suggest that VEGFR-1, but not VEGFR-2, is expressed significantly in nonmalignant cells and closely linked with CD68 infiltration “a M2 macrophage marker,” suggesting that it may have a greater role than VEGFR-2 in TME (Figure 3(a)). Additional studies support our own results implying that VEGFR-1 expression is higher in liver metastasis tissues than in primary tumours and thus is associated with tumour aggressiveness (Figures 3(a), 3(b), and 3(c)) [57, 58].

According to this, inflammatory mediators and cytokines such as the tumor necrosis factor (TNF)-*α*, transforming growth factor (TGF-*β*), and PlGF have previously been shown to have a significant effect on the inflammatory neovascularization implicating a prominent role of VEGFR-1 in this process [59, 60]. On the other hand, VEGFR-2 expression is mainly detected in malignant cells of primary CRC. VEGFR-1 but not VEGFR-2 expression is associated with poor survival time when analyzed alone in stages III and IV patients both in our unpublished data (Figure 3(c)) and literature data [20, 57, 58]. Altogether, our data and those reported in literature provide evidence that expression of VEGFR-1 is markedly associated with infiltration of inflammatory cells in the tumors [19, 20, 61, 62]. This is supported by the observation that PlGF potentiates the response to VEGF-A by signalling through VEGFR-1, and, in turn, it stimulates the recruitment of bone marrow derived macrophages to the tumour site by enhancing the expression of proangiogenic factors in the TME (Figure 2). PlGF exerts pleiotropic activities in the TME, by interacting specifically with VEGFR-1 on multiple types of vascular and nonvascular cells (Figure 2). Mechanisms of resistance or escape from anti-VEGF therapies (bevacizumab) may be promoted by releasing alternative angiogenic factors such as PlGF in malignant cells and inflammatory cells which might contribute to the induction of an angiogenic rescue program (Figure 2) [63].

## 4. Antiangiogenic Drugs in mCRC

In the last decade, different antiangiogenic strategies have been investigated in preclinical and clinical studies, showing how angiogenesis can be targeted by several approaches [64, 65]. Nowadays, it is possible to target angiogenesis with monoclonal antibodies which bind to VEGF, preventing its interaction with VEGFRs, with small molecules inhibiting tyrosine kinase (TK) activity of VEGFRs or using newer soluble receptor fragments like VEGF-trap technology (Figure 2) [66, 67]. Also anti-EGFR monoclonal antibodies cetuximab and panitumumab may have an indirect inhibition effect on angiogenesis [68]. Only three antiangiogenic agents have been approved by regulatory authorities for mCRC treatment: bevacizumab, regorafenib, and aflibercept [69].

### 4.1. Bevacizumab: Mechanism of Action and Clinical Studies

Bevacizumab, a humanized monoclonal antibody that binds to VEGF-A preventing its interaction with VEGFR-2, was the first antiangiogenic agent approved in mCRC (Figure 2 and Table 1). A randomized phase III trial comparing 5-fluorouracil/leucovorin and irinotecan (IFL) regimen alone or in combination with bevacizumab has shown that addition of antiangiogenic molecule improved not only RR (35 versus 45%) and PFS (6.2 versus 10.6 months) but also OS (15.6 versus 20.3 months) [12]. Also oxaliplatin based regimens have demonstrated survival benefit in combination with bevacizumab in both first- and second-line settings. In the NO16966 phase III trial, addition of bevacizumab to oxaliplatin based schedules with fluorouracil and leucovorin (FOLFOX) or capecitabine (XELOX) prolonged significantly PFS but OS difference was not statistically relevant. In this study, also response rates were similar between bevacizumab containing and not containing arms [70]. In second-line treatment for mCRC, the ECOG E3200 trial showed that addition of bevacizumab to FOLFOX4 regimen increased RR, PFS, and OS [71]. Recently, a meta-analysis of six randomized phase III studies showed that combination of bevacizumab to standard chemotherapy improves RR, PFS, and OS [72]. Bevacizumab main toxicities are peculiar and class specific and they are represented by arterial hypertension, bleeding, proteinuria, arterial or venous thrombosis, gastrointestinal perforation, and wound healing problems [70–72]. Another aspect of antiangiogenic treatment was recently investigated regarding the use of bevacizumab in patients who have progressed to a bevacizumab containing first-line therapy. The phase III randomized trial ML18147 evaluated the role of continuing bevacizumab plus standard chemotherapy after progression to bevacizumab plus chemotherapy first-line treatment. In this trial, patients who have received first-line bevacizumab containing therapy were randomized to receive second-line chemotherapy with alternate cytotoxic regimen with or without bevacizumab. Results obtained with this study show an advantage of maintaining bevacizumab plus chemotherapy beyond first progression, using an alternative cytotoxic regimen with significative prolongation in OS (11.2 versus 9.8 months; HR 0.81; 95% CI 0.69–0.84; *P* = 0.0062) and PFS (5.7 versus 4.1 months; HR 0.68; 95% CI 0.59–0.78; *P* < 0.0001) [73].

### 4.2. Regorafenib: Mechanism of Action and Clinical Studies

Regorafenib is an oral multikinase inhibitor with activity against selected tyrosine kinases (VEGFR-2 and -3, TIE-2, PDGFR, FGFR, RET, and c-Kit) as well as a signal transduction inhibitor of the RAF/MEK/ERK pathway (Figure 2 and Table 1) [74]. Regorafenib has been investigated as single agent in mCRC treatment after failure of standard therapy. The international, multicentered, randomized, double-blinded, placebo-controlled phase III trial CORRECT was conducted to evaluate efficacy of regorafenib in mCRC. In this study, 760 enrolled patients, who have progressed to all standard approved therapies, were randomized to receive regorafenib plus best supportive care (BSC) or placebo plus BSC. Primary end point of this study was OS. Preplanned interim analysis data showed a significantly longer OS in the regorafenib plus BSC arm than in the placebo plus BSC one (6.4 versus 5.0 months; HR 0.77; 95% CI 0.64–0.94; *P* = 0.0052). Also PFS was significantly prolonged by regorafenib (1.9 versus 1.7 months; HR 0.49; *P* < 0.000001). Response rate had no significative difference between two study arms (1% versus 0.4%), but regorafenib arm resulted in a higher disease control rate compared to placebo (45% versus 15%). Main toxicities related to this agent were represented by hand-foot skin reaction, fatigue, diarrhea, hypertension, and rash or desquamation [14].

## 5. Aflibercept: Trap-Technology and Peculiar Mechanism of Action

Aflibercept is a novel humanized recombinant fusion protein which acts as a decoy receptor binding to VEGF-A, VEGF-B, and PlGF resulting in the inhibition of their interaction with specific receptors (Figure 2 and Table 1). This molecule has been developed by employing the “trap technology” [75]. Aflibercept is a 97 kDA homodimeric glycoprotein deriving from the fusion of VEGFR-1 and VEGFR-2 extracellular domains to Fc portion of human IgG1 [76–78]. Aflibercept binds VEGF-A with high affinity with a 0.49 pMKd. In this way, aflibercept prevents interactions between VEGF and receptors exposed on cellular surface. Interestingly, aflibercept shows also high affinity for PlGF-2 with a Kd of 39 pM. Different from other antiangiogenic drugs, this mechanism of action should result in inhibition of MDSCs known to promote tumor progression and antagonize the antitumor efficacy (Figure 2). Anti-PlGF activity enhancing VEGFR inhibitor therapy should reduce the release of angiogenic factors by tumour and vascular cells (Figure 2). Aflibercept does not cause as much hypoxia and therefore does not induce an angiogenic escape programme as strongly as VEGF-VEGFR inhibitors do. This peculiar mechanism of action might reduce the incidence of drug resistance and explain, at least in part, the indication of aflibercept as valid second-line therapeutic option (Figure 3(d)).

### 5.1. Preclinical Studies

In vitro assays on cell lines evidenced that aflibercept blocks even VEGFR-2 mediated phosphorylation resulting in inhibition of endothelial cells proliferation necessary for new blood vessels formation [79]. Aflibercept significantly inhibits tumor growth and angiogenesis, reduces tumor vessel density, and inhibits metastases in xenografts of various tumor types [80–82]. When combined, in tumor xenografts, with other anticancer treatments such as cytotoxic chemotherapy or radiotherapy, aflibercept shows greater inhibition of tumor growth and vasculature than with the individual treatments alone. In particular, aflibercept has been investigated alone and in combination with cytotoxic chemotherapy in several murine models studies. In a 3-arm mammary adenocarcinoma study, aflibercept 40 mg/kg/dose twice weekly was as active as the highest nontoxic dose of 5-FU (90 mg/kg/dose) and the combination of two drugs showed synergistic activity at all tested doses. Single-agent aflibercept and irinotecan were equally active in mice with advanced-stage human colon cancer, with the combination of demonstrating synergy [83, 84]. Moreover, in tumor xenografts, aflibercept has shown decrease both in the expression of tumor vascular genes and in the activation of the vascular endothelial signalling pathways [85]. A dose-dependent effect has been observed in a xenograft model of neuroblastoma, whereas high doses of aflibercept led to greater regression of coopted vascular structures, which occurs during the initial phase of tumor growth [86]. Aflibercept has also shown activity on animal models in combination with several cytotoxic drugs such as docetaxel, paclitaxel, and gemcitabine or in combination with radiotherapy. It also showed synergistic activity with oxaliplatin, cisplatin, 5-fluorouracil, and S-1 [87–90]. Aflibercept activity has been observed at a dose ranging from 2.5 mg/kg to 40 mg/kg.

### 5.2. Phases I-II Clinical Trials

A phase I dose-escalation study of i.v. aflibercept in combination with irinotecan, infusional fluorouracil, and leucovorin showed that the recommended dose of aflibercept is 4 mg/kg i.v. every 2 weeks. This was an open-label, sequential cohort, and dose-escalation study. Primary end point was to find the dose limiting toxicity (DLT) during the first two cycles of treatment. Patients were treated with i.v. aflibercept over 1 hour on day 1 immediately followed by i.v. irinotecan 180 mg/m^2^ over 1 hour, then leucovorin 200 mg/m^2^ (or L-leucovorin 100 mg/m^2^) over 2 hours and 5-fluorouracil 400 mg/m^2^ i.v. bolus, and then 600 mg/m^2^ infusion over 22 hours on days 1 and 2 (Table 2). The first level dose of aflibercept was 2 mg/kg, and patients who did not experiment toxicities were enrolled to higher dose levels (4, 5, and 6 mg/kg). Thirty-eight patients affected by different type of cancer were enrolled. 61% of patients had mCRC. 36 had received previous chemotherapy, with 63% having previously received irinotecan. Adverse events associated with VEGF blockade were represented by mild to moderate hypertension (74%), dysphonia (74%), epistaxis (58%), and proteinuria (87%). Free aflibercept blood concentrations exceeded VEGF-bound aflibercept ones throughout the dose interval starting from 4 mg/kg. This observation suggested that aflibercept was administered at a biologically active dose [91]. Another phase I study investigated the safety dose limiting toxicities (DLTs) and recommended dose in 16 mCRC patients (Table 2). Two dose levels of aflibercept were assessed, 2 mg/kg and 4 mg/kg, respectively. DLTs had to be evaluated in the first two cycles. No DLTs were found. At recommended dose of 4 mg/kg, most common grade 3/4 toxicities were neutropenia 75% for both doses and hypertension in 25% at 4.0 mg/kg. The response rate and progression-free survival at 4.0 mg/kg were 8.3% and 7.59 months, respectively [92]. Aflibercept as monotherapy was investigated in two phase II trials (Table 2). In a phase II trial, 51 patients with mCRC who had received 1 or more lines of therapy were treated with aflibercept 4 mg/kg administered every 2 weeks. Primary end points were RR and 4 months PFS. 27 of 51 enrolled patients had received bevacizumab in the previous treatment regimens, while the remaining group was bevacizumab naïve. Disease control rate, defined as partial responses plus stable diseases, was 30% and 29%, while PFS was 3.4 and 2.0 months in bevacizumab pretreated and bevacizumab naïve patients, respectively. 7 patients in each group maintained 4-month PFS [93]. A more recent two-stage phase II study evaluated 75 mCRC patients treated with single-agent aflibercept at the dose of 4 mg/kg i.v. every 2 weeks. Patients were enrolled in two cohorts: bevacizumab naïve (24 patients) and prior bevacizumab (51 patients). Primary end point of this study was a combination of RR and 16-week PFS. The median PFS in the bevacizumab naıve and prior bevacizumab groups was 2.0 and 2.4 months, respectively. Aflibercept showed limited activity as a single agent ant it was well tolerated in pretreated mCRC, independent from prior therapy with bevacizumab [94]. Differently, the randomized phase 2 study AFFIRM investigated aflibercept combined with modified FOLFOX6 in first-line treatment of mCRC (Table 2). The primary end point was PFS rate at 12 months. The secondary end points included overall response rate, PFS, and OS. Preliminary results show no significant difference in PFS at 1 year [95].

### 5.3. The VELOUR Phase III Trial

The international, double-blinded, phase III trial VELOUR randomized 1,226 patients to receive either aflibercept 4 mg/kg i.v in combination with FOLFIRI or placebo every 2 weeks in combination with FOLFIRI after oxaliplatin treatment failure. Patients had adequate organ function and Eastern Cooperative Oncology Group performance status was 0 to 2. Median age was 61 years, 58.6% were men, and 56.4% had multiple metastatic sites. Approximately one-third of patients had previously been treated with bevacizumab. Primary end point was OS and secondary end points were PFS, RR, and also pharmacokinetics and immunogenicity. Addition of aflibercept to FOLFIRI significantly improved OS if compared to placebo plus FOLFIRI arm (12.06 versus 13.5 months; HR 0.817; 95.34% CI 0.713–0.937; *P* = 0.0032). PFS and response rate were also significantly improved. Median PFS was 6.90 months in the aflibercept arm and 4.67 months in the placebo arm (HR 0.758; 95% CI 0.661–0.869; *P* = 0.0001). Response rate was 19.8% (95% CI 16.4–23.2%) with aflibercept plus FOLFIRI versus 11.1% (95% CI 8.5–13.8%) with placebo plus FOLFIRI (*P* = 0.0001). Grades 3 and 4 adverse events with at least 2% higher incidence in aflibercept arm were represented by diarrhea, hypertension, asthenic conditions, neutropenia, proteinuria, stomatitis, ulceration, infections, gastrointestinal and abdominal pain, neutropenia, and neutropenic complications. Treatment discontinuation for toxicity occurred in 26.8% of patients receiving aflibercept plus FOLFIRI and 12.1% of patients receiving placebo [13]. A subset analysis evaluated differences in terms of outcome between patients who have progressed to an oxaliplatin plus bevacizumab therapy versus no prior bevacizumab treatment. This subgroups analysis found that the use of bevacizumab as a part of first line of therapy did not negatively affect clinical benefit of adding aflibercept to FOLFIRI [98] (Table 2). Given the results of VELOUR trial, aflibercept has been indicated for mCRC second-line treatment in addition to FOLFIRI in patients who have progressed to an oxaliplatin based first-line therapy independent from prior use of bevacizumab.

## 6. Concluding Remarks

Target agents have improved results obtained by standard chemotherapy in mCRC treatment and they have also enriched oncologists therapeutic weapons. Unfortunately, despite of these benefits, the prognosis of mCRC still remains poor as many patients experience disease progression after chemo- and target therapies. Today, the determination of Ras mutational status is mandatory before choosing the optimal strategy for treatment. Ras mutant status suggests that using anti-EGFR agents in these patients is not only ineffective but, in some cases, could be detrimental. On the other hand, antiangiogenic therapies could be proposed to every patient according to his performance status, comorbidities, and clinical conditions, because of the lack of biomarkers and predictive factors of response which may help us to better select patients for treatment with these molecules. Antiangiogenic agent bevacizumab has been approved in both first- and second-line treatment of mCRC. Clinical trials suggest that antiangiogenic treatment with bevacizumab could be continued also beyond first progression, and newer agents such as regorafenib and aflibercept have recently been approved in mCRC third- and second-line therapy, respectively. This evidence enforces the concept that targeting CRC through angiogenesis inhibition also after failure of a first antiangiogenic treatment could be equally successful. An important question to point out is about the optimal antiangiogenic strategy to use after a bevacizumab based first-line therapy. In this scenario, aflibercept seems to be an optimal molecule to target angiogenesis. Addition of aflibercept to FOLFIRI in second-line treatment for mCRC allowed a 1.4-month gain in OS that is identic to OS prolongation reached by bevacizumab in second-line treatment in ML18147 study. Given toxicity profile and not significantly higher survival benefit of aflibercept in second-line therapy if compared with bevacizumab beyond first progression, one could conclude that aflibercept is not an ideal agent for this setting. Nevertheless, aflibercept has a peculiar and unique mechanism of action that is different from other antiangiogenic agents. In fact, whereas bevacizumab targets angiogenesis through VEGF-A inhibition, aflibercept inhibits VEGF-A, -B and PlGF signaling pathways. This mechanism of action should allow the blockage of tumor progression at different levels acting on malignant cells, macrophages, and stromal cells that contribute with cooperative mechanisms to tumour neovascularization. Therefore, using aflibercept in second-line therapy in patients who have progressed to bevacizumab may help (1) to overcome resistances, (2) limit the compensatory angiogenic factors, and (3) modulate the activity of inflammatory cells within the tumor microenvironment. This concept is consistent with clinical evidences that VEGF-A inhibition, in bevacizumab treated patients, is associated with VEGFR-2 and PlGF increased levels. In this way, PlGF contributes to the induction of an angiogenic rescue program while VEGF-A is blocked, thus contributing to tumor escape to bevacizumab action. This suggests that aflibercept could be active in avoiding tumor escape mechanism and restore sensitivity to antiangiogenic treatment (Figure 3(d)). Moreover, given the crucial role of PlGF/VEGFR-1 axis in inflammation, neovascularization, and tumor aggressiveness, the use of aflibercept could be helpful to target both proangiogenic factors and the inflammatory cells surrounding the tumor microenvironment.

Aflibercept has improved OS, PFS, and RR in second-line therapy for mCRC in addition to FOLFIRI regimen after failure of a first-line oxaliplatin containing regimen. Actually, this new molecule can be considered a standard of care in this setting both in Ras wild-type and mutant patients and it can be successfully used after anti-EGFR molecules as well as after bevacizumab treatment. Its peculiar mechanism of action may help to overcome tumor escape mechanisms to bevacizumab treatment. Nowadays, we have no biomarkers of response to aflibercept that may help us to candidate patient to this therapy. Further studies are required to better investigate the role of aflibercept in the contest of a global strategy of treatment for mCRC.

## Figures and Tables

**Table 1:** Bevacizumab, aflibercept, and regorafenib are antiangiogenic drugs currently approved for mCRC treatment. These drugs have proved successful for the clinical treatment of various types of cancer and their mechanism of action results different affecting a variable range of cell types and signaling within tumor microenvironment.

Drug	Indication	Mechanism of action
Bevacizumab	First-/second-line mCRC plus CT	MoAb binding VEGF-A
Aflibercept	Second-line mCRC plus FOLFIRI	VEGF decoy binding VEGF-A, VEGF-B, and PlGF
Regorafenib	Third-line mCRC as single agent	Multikinase inhibitor of RTKI of VEGFR-2 and -3, TIE-2, PDGFR, FGFR, RET, and c-kit

**Table 2:** Aflibercept in mCRC has been clinically developed in several phases I-II clinical trials and in a final phase III randomized trial. This table briefly summarizes results of efficacy, activity, and safety in published/presented clinical trials with aflibercept in mCRC.

Study	Phase	Setting	Patients (num.)	Aflibercept schedule	Efficacy	Safety (grade 3/4 toxicities)
Yoshino et al. [92] Invest New Drugs 2013 Aug; 31 (4): 910–7	I	mCRC	16	2–4 mg/kg + FOLFIRI	ORR (*n* = 13 at 4 mg/kg ): 7.7% SD: 69.2% Median PFS: 7.6 mo	Neutropenia: 75% Hypertension: 25%

Verslype et al. [96] ASCO 2008 Abstract (14540)	I	Recurrent solid tumors (70% CRC)	27	4 mg/kg or PBO + FOLFIRI Cy 1 → 4 mg/kg + FOLFIRI q2 wk	ORR: 15.4% SD: 65.4%	Neutropenia: 37.0% Fatigue: 29.6% Hypertension: 29.6% Dyspnea: 22.2%

van Cutsem et al. [91] Eur J Cancer. 2013; 49: 17–24	I	Recurrent solid tumors (61% CRC)	38	2, 4, 5, and 6 mg/kg, and FOLFIRI	Not a study end point	**Serious AEs in 27 patients ** 50%, 67%, 70%, and 58% of patients at aflibercept 2, 4, 5, and 6 mg/kg, respectively

Tang et al. [93] J Clin Oncol 2008; 26 (suppl): abstract 4027	II	Previously treated mCRC	51	4 mg/kg q2wk monotherapy	DCR 30% vs 29% PFS 3.4 vs 2.0 months (bev. pretreated vs naïve)	Hypertension, proteinuria 8% fatigue, headache 6%

Tang et al. [94] Clin Cancer Res 2012; 18: 6023–31	II	Recurrent CRC	75	4 mg/kg q2wk monotherapy	PFS 2.4 vs 2.0 months (bev. pretreated vs naïve)	Hypertension 9.1% Proteinuria 9% Fatigue, headache 5%

AFFIRM Pericay et al. [95] Ann Oncol 2012; 23 (Suppl. 4) Abstract 0024	II Ran	Untreated mCRC	235	4 mg/kg ± mFOLFOX6 q2wk	12 months PFS rate: 25.8% vs 21.2% (aflibercept + mFOLFOX6 vs mFOLFOX6)	**Grade 3/4 toxicities > 5% higher in aflibercept arm ** Hypertension, neutropenia, diarrhea Infection

Van Cutsem et al. [97] J Clin Oncol 2012; 30: 3499–506	III Ran	Oxaliplatin pretreated mCRC	1226	4 mg/kg ± FOLFIRI q2wk	OS = 13.50 vs 12.06 months PFS: 6.90 vs 4.67 months ORR: 19.8% vs 11.1% (aflibercept + FOLFIRI vs FOLFIRI)	Diarrhea 19.3% Neutropenia 36.7% Stomatitis and ulceration 13.7% Hypertension 19.3% Proteinuria 7.9% TVE 7.9%

bev.: bevacizumab; DCR: disease control rate; mCRC: metastatic colorectal cancer; mFOLFOX6: modified FOLFOX6; num.: number; ORR: objective response rate; OS: overall survival; PBO: placebo; PFS: progression-free survival; q2wk: every 2 weeks; Ran: randomized; SAEs: severe adverse events; SD: stable disease; TVE: thromboembolic venous events; vs: versus.
